# PP91 Is The Creation Of A Unified Health Technology Assessment Agency In Brazil A Good Idea?

**DOI:** 10.1017/S0266462325103085

**Published:** 2025-12-29

**Authors:** Elene Paltrinieri Nardi, Ricardo Rodrigues Teixeira, Daniela Melo

## Abstract

**Introduction:**

In Brazil, decisions regarding the incorporation of health technologies, guided by health technology assessment (HTA), are made independently within the public and private health systems. There is ongoing discussion about establishing a unified HTA agency to address incorporation across both sectors. This study sought to compare the decision-making criteria for technology incorporation in Brazil’s public and private health systems.

**Methods:**

A dataset of new medicines registered in Brazil between 2010 and 2020, along with their corresponding indications, was compiled to identify and assess medication-indication pairs. Case studies were developed for pairs that underwent coverage evaluations in both the public and private health systems. These HTA coverage decisions were systematically analyzed and compared using a published evidence-based methodological framework. This methodology requires that research questions from different HTA agencies be sufficiently alike to enable a meaningful comparison of decision-making criteria.

**Results:**

Twenty medicines across 10 indications were analyzed. Oncology related pairs (10 medicines, five indications) could not be compared due to differing research focuses: public evaluations covered entire treatment lines, whereas private assessments focused on specific medicine-indication pairs. Non-oncology cases (10 medicines, five indications) targeted eosinophilic asthma, ulcerative colitis, psoriasis, multiple sclerosis, and neuronal ceroid lipofuscinosis type 2. Public sector decisions prioritized unmet needs, disease impact, clinical benefits, and negotiated prices. In contrast, the private sector focused on expanding therapeutic options, with clinical uncertainties having limited influence. The private sector did not consider costs in decision-making, even when medicines lacked clinical superiority over comparators.

**Conclusions:**

Decision-making in the public sector relies on HTA principles, while the private sector focuses on therapeutic expansion, ignoring efficiency and clinical uncertainties. A unified HTA agency in Brazil could negatively affect the public sector if private sector priorities dominate. Moreover, such an agency is unlikely to reduce the workload, as research questions differ significantly across sectors.

